# Mechanical, Durability and Microstructural Performance of OPC–GGBFS–FGD Gypsum Ternary Concrete: Identification of an Operational Sulfate Activation Threshold

**DOI:** 10.3390/ma19142962

**Published:** 2026-07-09

**Authors:** Anand Bhatt, Sanjay Kumar, Prahlad Prasad, Anasuya Sahu, Pramod Kumar, Ardalan B. Hussein

**Affiliations:** 1 Department of Civil Engineering, National Institute of Technology Jamshedpur, Jamshedpur 831014, India; 2 Department of Civil Engineering, Nirma University, Ahmedabad 382481, India; 3 Department of Civil Engineering, Graphic Era Deemed to be University, Dehradun 248002, India; 4 Department of Structural Engineering and Geotechnics, Széchenyi István University, Egyetem Tér 1, 9026 Győr, Hungary

**Keywords:** chloride penetrability, flue gas desulfurization gypsum, ground granulated blast-furnace slag, microstructural analysis, strength efficiency index, sulfate activation, ternary blended concrete

## Abstract

Ordinary Portland cement (OPC) production contributes approximately 7–8% of global anthropogenic CO_2_ emissions, driving urgent demand for clinker-efficient binders utilizing industrial by-products. Flue gas desulfurization (FGD) gypsum and ground granulated blast-furnace slag (GGBFS) represent underutilized industrial by-products with documented potential as supplementary cementitious materials. This study investigates the mechanical, durability and microstructural performance of OPC–GGBFS–FGD gypsum ternary concrete mixtures incorporating untreated flue gas desulfurization (FGD) gypsum at 0–20% of total binder mass and ground granulated blast-furnace slag (GGBFS) at 25–50% of total binder mass in M30 structural concrete (w/b = 0.45). Compressive, split tensile and flexural strengths were evaluated at 7–90 days alongside rapid chloride penetration (RCPT), water absorption, strength efficiency index (SEI) and SEM–EDX analyses. Binary GGBFS replacement progressively enhanced long-term compressive strength, with T35F0 attaining 55.6 N/mm^2^ at 90 days (+33.7% relative to the OPC control). Moderate FGD gypsum contents (5–10%) further enhanced overall performance. Among all mixtures, T50F10 exhibited the best overall performance on the mechanical and durability indicators evaluated, achieving 54.2 N/mm^2^ compressive strength at 90 days together with a rapid chloride permeability value of 410 C, corresponding to ‘Very Low’ chloride ion penetrability. Beyond 10% FGD gypsum, progressive multi-parameter deterioration was observed, and mixtures containing 20% FGD gypsum failed to meet the M30 design requirement at 28 days. SEM–EDX confirmed that optimum sulfate activation produced a dense C–(A)–S–H-rich matrix, while excess sulfate caused matrix disruption. The findings establish 10% FGD gypsum by total binder mass as the optimum sulfate activation threshold for the investigated GGBFS and FGD gypsum sources at w/b = 0.45, and demonstrate the potential of untreated industrial FGD gypsum to produce durable, low-clinker structural concrete.

## 1. Introduction

Ordinary Portland cement (OPC) production exceeds 4.1 billion tons annually, contributing approximately 7–8% of global anthropogenic CO_2_ emissions [1,2,3]. The escalating infrastructure demands of developing economies, combined with tightening carbon reduction commitments, have accelerated the adoption of supplementary cementitious materials (SCMs) as the principal near-term strategy for clinker substitution [4,5,6]. Ground granulated blast-furnace slag (GGBFS) and calcium sulfate-bearing by-products are among the most technically mature and volumetrically significant SCMs available to the construction sector [5,7,8].

GGBFS exhibits latent hydraulic reactivity upon alkaline or sulfate stimulation, generating calcium (alumino-)silicate hydrate gels with superior pore-filling capacity and long-term strength development relative to OPC hydration products [5,8,9]. Binary OPC–GGBFS concrete systems consistently improve chloride resistance, reduce heat of hydration and extend service life in aggressive environments [5,9,10]. Nevertheless, effective slag activation requires adequate availability of both hydroxyl and sulfate ions; without sufficient activation, early-age strength development in slag-rich systems may be suboptimal for structural applications [8,11].

Flue gas desulfurization (FGD) gypsum, a CaSO_4_·2H_2_O by-product of wet SO_2_ scrubbing in coal-fired power generation, is produced globally at 200–250 million tons per year [12,13,14]. In India, annual FGD gypsum output already exceeds 30 million tons and is growing as new SO_2_ regulations mandate the installation of scrubbers across the thermal power fleet [12,15]. A significant fraction of this material is currently landfilled, creating environmental liabilities and representing an unrealized resource for the construction industry [13,16]. Its high CaSO_4_·2H_2_O content and fine particle size distribution makes FGD gypsum a candidate sulfate activator for GGBFS-rich binder systems, capable of simultaneously reducing solid waste disposal burdens and OPC clinker consumption.

Prior investigations have collectively demonstrated that moderate sulfate additions promote C–(A)–S–H gel refinement, pore structure densification and improved interfacial transition zone quality in slag-blended systems, yielding compressive strength gains of 10–25% and substantial reductions in chloride penetrability relative to equivalent binary blends [5,7,9,17]. The governing mechanism involves SO_4_^2−^-driven dissolution of the amorphous GGBFS glass network and accelerated consumption of aluminate phases, with concurrent formation of ettringite and C–(A)–S–H at moderate dosages. However, this activation is strongly dose-dependent. Excessive sulfate supply disrupts hydration equilibrium, promotes deleterious ettringite expansion, and leads to progressive strength deterioration beyond a composition-specific critical threshold [15,18,19]. Establishing this threshold precisely, as well as validating it across a full suite of mechanical and durability metrics, remains an open research question.

Despite this body of evidence, several gaps limit the translation of laboratory findings to engineering practice. Most published studies have employed laboratory-grade or thermally pretreated gypsum rather than as-received industrial FGD by-product material, whose trace impurity content and mineralogical variability differ from those of purified sources [12,13]. Systematic dose–response studies spanning the full practical range of FGD gypsum contents (5–20% of GGBFS mass) across multiple slag replacement levels remain scarce; most investigations evaluate one or two fixed sulfate additions, precluding reliable threshold identification [7,20,21,22]. Comprehensive simultaneous assessment of compressive, split tensile and flexural strength alongside rapid chloride penetration and water absorption, at 28, 56 and 90 days, is likewise uncommon in the FGD gypsum literature, as is the use of the strength efficiency index (SEI) as a clinker utilization metric. Furthermore, published data from the Indian industrial context, where GGBFS from integrated steel plants and FGD gypsum from thermal power stations represent co-located, high-volume industrial streams, are particularly limited, constraining the regional applicability of existing guidance.

The present study addresses these gaps through a systematic experimental program that incorporates untreated FGD gypsum sourced from NTPC Singrauli Super Thermal Power Station and GGBFS from Tata Steel Limited, Jamshedpur, into M30 structural concrete across 13 mix compositions. The specific objectives are: (i) to characterize the fresh, mechanical and durability performance of OPC–GGBFS–FGD gypsum ternary concrete across GGBFS replacement levels of 25–50% and FGD gypsum dosages of 0–20% of total binder mass; (ii) to identify, for the investigated GGBFS and untreated FGD gypsum sources, the operational FGD gypsum threshold beyond which mechanical and durability performance undergoes consistent multi-parameter deterioration; (iii) to quantify clinker utilization efficiency through SEI analysis; (iv) to establish the mechanistic basis for observed performance trends through SEM–EDX microstructural characterization and Ca/Si ratio analysis of hydration gel phases at 90 days.

The novelty of this work is threefold. Among the limited investigations employing industrially sourced, as-received FGD gypsum, this study is one of the few to conduct a full dose–response characterization across thirteen compositional variants from a major Indian steel–power supply chain. The simultaneous identification of a precise operational sulfate threshold (10% FGD gypsum by total binder mass) for the investigated material system, validated across six performance metrics from 7 to 90 days, provides a more complete compositional map than has been previously reported for comparable systems. Third, EDX-derived Ca/Si ratios and SEI values are jointly interpreted to link the character of the hydration gel with clinker utilization efficiency, thereby connecting microstructural evidence to a practical design criterion. The findings provide a transferable framework for the large-scale valorization of co-located industrial by-products in durable, low-clinker structural concrete.

## 2. Experimental Program

### 2.1. Raw Materials and Physicochemical Characterization

Ordinary Portland cement (OPC, 53 grade) conforming to IS 12269:2013 [23] served as the primary binder. Ground granulated blast-furnace slag (GGBFS) procured from Tata Steel Limited (Jamshedpur) and satisfying IS 16714:2018 [24] was incorporated as the latently hydraulic supplementary cementitious material (SCM). Untreated flue gas desulfurization (FGD) gypsum collected from the NTPC Singrauli Super Thermal Power Station was used as a sulfate-bearing mineral additive in its as-received condition, without thermal pre-treatment. The use of untreated FGD gypsum is supported by previous studies reporting its mineralogical similarity to natural gypsum, with calcium sulfate dihydrate (CaSO_4_·2H_2_O) as the predominant crystalline phase [11,13]. Natural river sand (Zone II) and crushed granite coarse aggregate (nominal maximum size 20 mm) conforming to IS 383:2016 [25] were used as fine and coarse aggregates, respectively. A polycarboxylate ether-based superplasticizer (PCE-SP) conforming to ASTM C494 Type F [26] was incorporated at a constant dosage of 0.5% by binder mass (1.9 kg/m^3^) across all mixtures to maintain adequate workability without altering the water-to-binder ratio. Potable water satisfying the quality requirements of IS 456:2000 [27] was used throughout the investigation.

**Figure 1:**
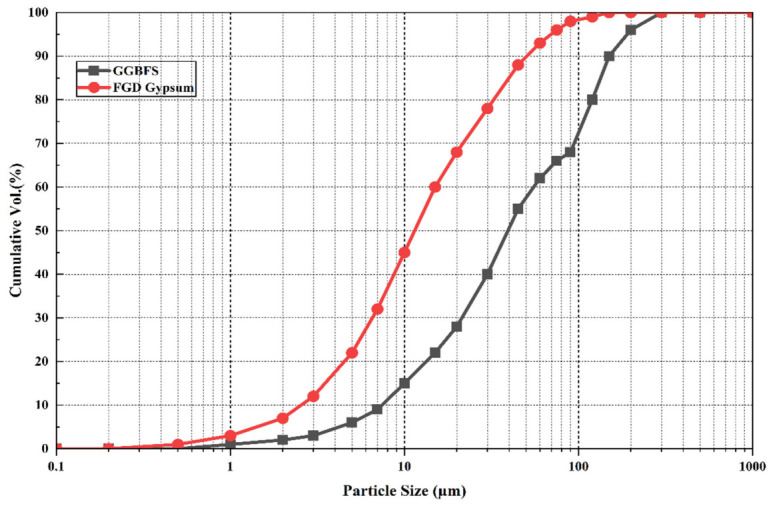
Particle size distribution curves of GGBFS and FGD gypsum. FGD gypsum exhibited a finer particle size distribution, indicating higher specific surface area and reactivity.

Physicochemical characterization of all cementitious materials was performed prior to mix proportioning. Physical and particle-size properties are summarized in Table 1, while the chemical oxide compositions, obtained from supplier datasheets and corroborated with published literature for similar material systems [5,11,12,19], are also presented in the same table. Particle size distributions (PSD) of GGBFS and untreated FGD gypsum were determined using laser diffraction analysis, whereas OPC reference values were adopted from IS 12269:2013 [23] and published characterization studies. The corresponding PSD curves are presented in Figure 1.

**Figure 2:**
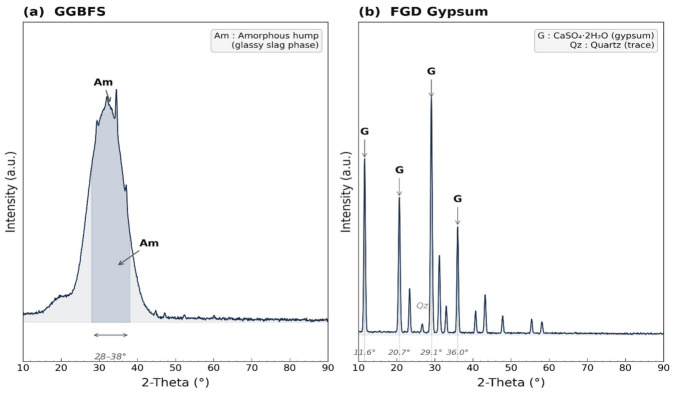
XRD patterns of (**a**) GGBFS and (**b**) untreated FGD gypsum. GGBFS shows a broad amorphous hump (28–38° 2θ), indicating a glassy phase; FGD gypsum shows sharp crystalline peaks of CaSO_4_·2H_2_O with trace quartz.

Mineralogical characterization of GGBFS and untreated FGD gypsum was carried out using X-ray diffraction (XRD) analysis with Cu-Kα radiation over a 2θ scanning range of 10–80°. The corresponding diffraction patterns are presented in Figure 2. The GGBFS pattern exhibited a broad diffuse hump between approximately 28° and 38°, indicating the predominantly amorphous vitreous nature of slag phases, whereas untreated FGD gypsum showed distinct crystalline peaks primarily corresponding to calcium sulfate dihydrate phases together with minor impurity peaks associated with desulfurization by-products. The finer particle distribution of GGBFS and untreated FGD gypsum contributes to improved particle packing, enhanced filler effects, and modified hydration evolution within the ternary binder system.

The untreated FGD gypsum used in this study exhibited a loss on ignition of 12.74%, substantially higher than that of OPC (1.96%) and GGBFS (1.42%) (Table 1), reflecting residual moisture, carbonate impurities and minor organic content characteristic of as-received wet flue gas desulfurization by-products. The measured SO_3_ content of 43.85% corresponds to approximately 95% CaSO_4_·2H_2_O purity relative to the theoretical SO_3_ content of pure gypsum (≈46.5%), with the remainder attributable to trace quartz and other minor desulfurization by-product phases, consistent with the impurity peaks observed in the XRD pattern (Figure 2b). Source-to-source variability in FGD gypsum composition, arising from differences in coal feedstock, scrubber limestone purity, and flue gas chemistry, is well documented across thermal power stations [12,13,14]; the impurity profile characterized here is specific to the NTPC Singrauli source, and re-characterization would be required prior to application of untreated FGD gypsum from other sources.

### 2.2. Mixture Proportioning

All thirteen mixtures maintain a constant total binder content of 380 kg/m^3^ and a constant water-to-binder ratio of 0.45 (water = 171 kg/m^3^). GGBFS content is fixed at the T-level percentage of total binder (e.g., T50 → GGBFS = 190 kg/m^3^ = 50% × 380 kg/m^3^). FGD gypsum is introduced at the F-level percentage of total binder (e.g., F10 → FGD gypsum = 38 kg/m^3^ = 10% × 380 kg/m^3^). OPC fills the remaining binder content (OPC = 380 − GGBFS − FGD gypsum). Consequently, increasing FGD gypsum content displaces OPC within a constant total binder system; the GGBFS content at any given T-level is unchanged across all F-levels. This design isolates the effect of sulfate availability on hydration and transport behavior while preserving constant paste volume and w/b ratio across all compositions.

On this basis, a total of 13 concrete mixtures were designed to investigate the influence of incorporating GGBFS and untreated FGD gypsum on the mechanical, durability, and microstructural performance of structural concrete. The control mixture (T0F0) was prepared using OPC as the sole binder, with GGBFS replacement levels of 25–50% to evaluate slag hydration behavior under conventional curing conditions.

Untreated FGD gypsum contents ranging from 5 to 20% of total binder mass were incorporated into selected slag-rich mixtures to investigate the influence of sulfate availability on hydration evolution and transport resistance. Particular emphasis was placed on identifying the operational sulfate threshold beyond which deterioration in mechanical and durability performance occurs. The water-to-binder ratio and aggregate proportions were maintained constant for all mixtures to isolate the influence of binder modification.

**Figure 3:**
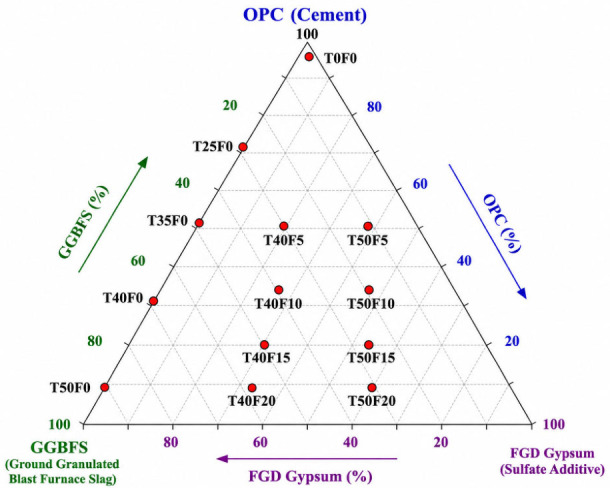
Ternary composition diagram showing binder proportions of OPC, GGBFS and FGD gypsum. T0F0 (control) was 100% OPC; binary and ternary systems were developed by partially replacing OPC with GGBFS and varying FGD gypsum content (0–20% of total binder mass).

Concrete mixture design was performed in accordance with IS 10262:2019 [28] for M30-grade concrete. The nomenclature adopted for the mixtures represents the replacement level of GGBFS (T) as a percentage of the total binder mass and the FGD gypsum content (F) as a percentage of the total binder mass, both substituted for OPC within a constant total binder content of 380 kg/m^3^. The binder compositions of all thirteen mixtures are presented in the ternary diagram in Figure 3, and the corresponding proportions by mass are listed in Table 2.

### 2.3. Specimen Preparation and Curing

Dry mixing of OPC, GGBFS, untreated FGD gypsum, and aggregates was performed in a pan mixer for 2 min to ensure uniform distribution of the constituent materials. Water and superplasticizer were subsequently introduced, followed by wet mixing for an additional 3 min until homogeneous concrete was obtained.

Fresh concrete was cast into molds in two layers and compacted using a table vibrator. Cube specimens (150 × 150 × 150 mm) were prepared for compressive strength testing [29], cylindrical specimens (150 × 300 mm) for split tensile strength testing [30], and prism specimens (100 × 100 × 500 mm) for flexural strength testing [31]. Disk specimens with a diameter of 100 mm and a thickness of 50 mm were prepared for RCPT measurements [32].

Following casting, specimens were covered with polyethylene sheets for 24 h to minimize moisture loss. After demolding, all specimens were cured under water at 27 ± 2 °C until the designated testing ages.

### 2.4. Testing Methods

Compressive strength was determined on 150 mm cube specimens at 3, 7, 28, 56 and 90 days in accordance with IS 516 [29]. Split tensile strength was evaluated on 150 × 300 mm cylindrical specimens at 28, 56 and 90 days in accordance with IS 5816 [30]. Flexural strength was determined on 100 × 100 × 500 mm prism specimens under third-point loading at 28, 56 and 90 days in accordance with IS 516 [31]. All mechanical tests were conducted on three replicate specimens and results are reported as arithmetic means with ±1 SD. Water absorption was determined in accordance with ASTM C642 [33] at 28 and 90 days; oven-dried specimens were immersed until saturation and absorption was calculated from the difference between saturated and dry masses. Rapid chloride permeability testing was conducted in accordance with ASTM C1202 [32] on 100 mm diameter disk specimens of 50 mm thickness at 28, 56 and 90 days; a constant potential difference of 60 V was applied for 6 h and chloride penetrability was classified according to the thresholds specified in ASTM C1202. The loading rate during all mechanical tests was maintained within the limits specified by the corresponding standards.

### 2.5. Strength Efficiency Analysis

The strength efficiency index (SEI) was used to evaluate clinker utilization efficiency in the investigated concrete mixtures. SEI was determined as the ratio between compressive strength and cement content in the corresponding mixture:

$$SEI=\frac{f_{c,mix}/C_{mix}}{f_{c,OPC}/C_{OPC}}$$

where $f_{c,mix}$ is the compressive strength of the blended concrete mixture at a given curing age, $C_{mix}$ is the cement content of the corresponding mixture, $f_{c,OPC}$ is the compressive strength of the OPC control mixture at the same curing age, and $C_{OPC}$ is the cement content of the control mixture.

### 2.6. Statistical Treatment

The statistical framework adopted for the analysis of experimental data in this study is summarized in Table 3. All mechanical and durability test results are reported as arithmetic means of three replicate specimens (n = 3) with ±1 standard deviation (SD). Coefficients of variation (CoV) ranged from 1.33% (T50F10, 90 days) to 2.60% (T40F20, 14 days) across all 65 compressive strength test groups, with a mean CoV of 1.85%, confirming acceptable experimental reproducibility. CoV decreased with curing age within each mixture and was systematically higher in mixtures containing FGD gypsum contents above the optimum threshold, consistent with the matrix heterogeneity described in Section 3.2 and Section 3.7. Outliers were identified and excluded using Grubbs’ criterion (α = 0.05); no outliers were detected in any test group.

## 3. Results and Discussion

### 3.1. Fresh Properties of Concrete

**Figure 4:**
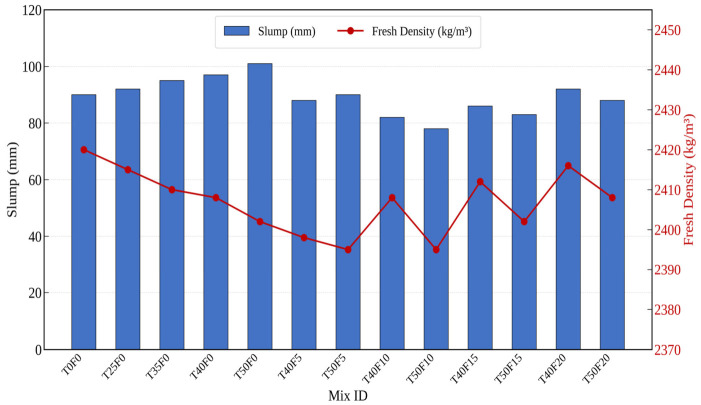
Fresh properties of all thirteen mixtures: slump (bars, left axis, per IS 7320:1974) and fresh density (line, right axis). Shaded regions indicate compositional zones: binary GGBFS (T0F0–T50F0), ternary FGD gypsum (T40F5–T50F15), and detrimental (T40F20–T50F20).

The slump values of all concrete mixtures ranged from 78 to 101 mm (Figure 4), indicating workability suitable for normal structural concrete applications. The OPC control (T0F0) recorded a slump of 90 mm, while binary GGBFS mixtures showed a marginal increase at moderate replacement levels, with T50F0 attaining the highest slump of 101 mm. A fixed dosage of polycarboxylate ether-based superplasticizer was maintained constant across all mixtures to isolate the effect of binder composition on workability; the observed slump increase in binary GGBFS mixtures is therefore attributable to the inherent surface characteristics of the slag rather than to any variation in chemical admixture dosage. This improvement may be attributed to the comparatively smooth particle surface texture of vitreous slag, which reduces interparticle friction in the fresh state and increases the availability of free water [5,21]. Progressive addition of FGD gypsum consistently reduced workability at both 40% and 50% GGBFS replacement levels; T50F10 was 78 mm, representing a 12 mm reduction relative to the control. This reduction is attributed to the finer particle size distribution of FGD gypsum (D_50_ = 11.7 µm) and its elevated surface area, which increases the water demand of the mixture and reduces the proportion of free water available to lubricate particle motion, an effect that was not offset by the constant PCE-SP dosage [4,5,34]. All mixtures exhibited workable consistency suitable for structural concrete applications.

The fresh density of all mixtures varied within a narrow range of 2395–2420 kg/m^3^ (Figure 4), with values declining progressively as SCM content increased, attributable to the lower specific gravities of GGBFS (2.70) and FGD gypsum (2.04) relative to OPC (3.09). The control mixture (T0F0) recorded the highest fresh density at 2420 kg/m^3^. The maximum deviation from the control was 25 kg/m^3^ (1.0%), a difference of no practical significance for structural design purposes, and confirms that the combined substitution of OPC with GGBFS and FGD gypsum does not materially alter the unit weight of fresh concrete within the studied replacement range [19,21].

### 3.2. Compressive Strength Development

**Figure 5:**
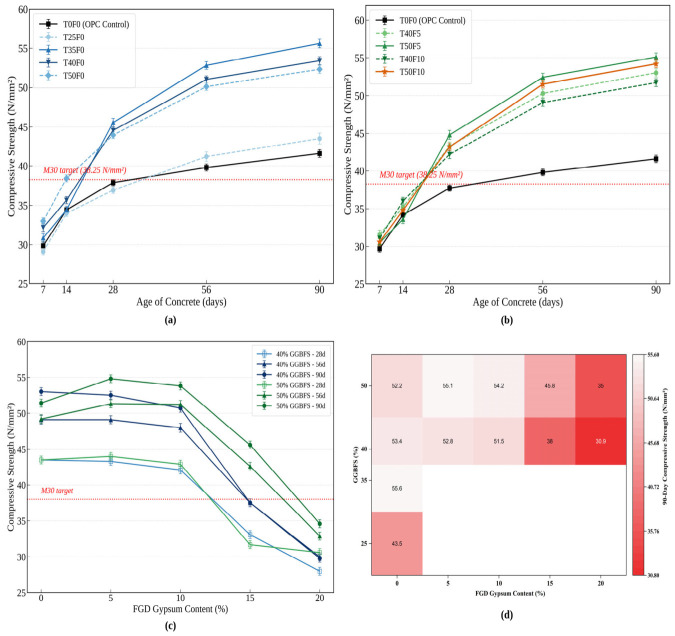
Compressive strength development and compositional response of OPC–GGBFS–FGD gypsum concrete mixtures: (**a**) binary GGBFS blends, (**b**) ternary mixtures containing moderate FGD gypsum contents (5–10%), (**c**) influence of FGD gypsum dosage at 40% and 50% GGBFS replacement levels, and (**d**) heatmap representation of 90-day compressive strength distribution.

The compressive strength results of all mixtures at different curing ages are presented in Figure 5a–c, while Figure 5d presents the heatmap distribution of 90-day compressive strength for all investigated mixtures. Figure 5a–c illustrate the strength development trends with curing age for the binary and ternary OPC–GGBFS–FGD gypsum systems, whereas Figure 5d provides a comparative compositional overview of the long-term compressive strength response at 90 days. The binary GGBFS replacement series exhibited no significant reduction in early-age strength, as all F0 mixtures achieved 7-day compressive strengths comparable to that of the OPC control mixture, T0F0 (29.9 N/mm^2^). Beyond 14 days, all binary blends progressively exceeded the strength of the control mixture due to the continued latent hydraulic and pozzolanic reactions of GGBFS. As shown in Figure 5a, T35F0 achieved the highest 90-day compressive strength of 55.6 N/mm^2^, corresponding to an improvement of approximately 33.7% relative to T0F0 (41.6 N/mm^2^). T40F0 and T50F0 also exhibited high 90-day strengths of 53.4 and 52.3 N/mm^2^, respectively, whereas T25F0 attained 43.5 N/mm^2^.

The 28-day compressive strength results presented in Figure 5d further confirmed that all binary mixtures satisfied the target mean strength requirement for M30 concrete. The long-term strength enhancement is attributed to the gradual formation of secondary calcium silicate hydrate (C–S–H) gel generated through the reaction of GGBFS with calcium hydroxide released during OPC hydration [5,21]. However, a slight reduction in compressive strength beyond 35% GGBFS replacement, particularly in T50F0, suggests that excessive slag replacement may reduce the availability of Ca(OH)_2_ required for effective slag activation [5,8,21,35].

The incorporation of moderate FGD gypsum contents (5–10%) further improved compressive strength relative to both the OPC control and the corresponding binary blends, as illustrated in Figure 5b. Among all ternary mixtures, T50F5 achieved the highest 90-day compressive strength of 55.1 N/mm^2^, closely followed by T50F10 with 54.2 N/mm^2^ (+1.6% difference). Despite this marginally higher compressive strength and SEI (1.270 vs. 1.250, +1.6%), T50F10 is identified as the optimum composition on the basis of its superior performance on the remaining four of six evaluated metrics: split tensile strength (4.25 vs. 4.05 N/mm^2^, +4.9%), flexural strength (5.82 vs. 5.60 N/mm^2^, +3.9%), RCPT (410 C vs. 524.62 C, a 21.8% reduction in charge passed) and water absorption (2.92% vs. 3.06%, a 4.6% reduction), as demonstrated in subsequent sections. The compressive strength and SEI differences between T50F5 and T50F10 are modest and within the range of typical experimental variability (CoV 1.3–2.6%; Section 2.4), whereas the durability advantage of T50F10, particularly its substantially lower chloride penetrability, is of direct relevance to structural performance in aggressive service environments. The dose–response behavior shown in Figure 5c clearly identifies the 5–10% FGD gypsum range as the optimum compositional zone for both 40% and 50% GGBFS replacement levels. The observed enhancement is associated with sulfate-assisted activation of slag hydration, in which sulfate ions released from FGD gypsum promote additional hydration reactions and improve interfacial transition zone (ITZ) densification by increasing the formation of secondary C–S–H gel [5,20,34,36].

Beyond the optimum gypsum content of 10%, compressive strength decreased progressively at all curing ages, as shown in Figure 5c. The mixtures T40F20 and T50F20 recorded the lowest 90-day strengths of 30.9 and 35.0 N/mm^2^, respectively. In addition, the 28-day strengths of T40F15 and T40F20 fell below the target M30 strength level indicated in Figure 5d. This reduction in performance is primarily attributed to excessive clinker dilution and sulfate imbalance, which adversely affect hydration stability and matrix continuity. The trend is consistent with the SEM–EDX observations discussed in Section 3.7, in which excessive gypsum content resulted in increased porosity and microstructural disruption [15,19].

### 3.3. Failure Characteristics and Fracture Surface Analysis

**Figure 6:**
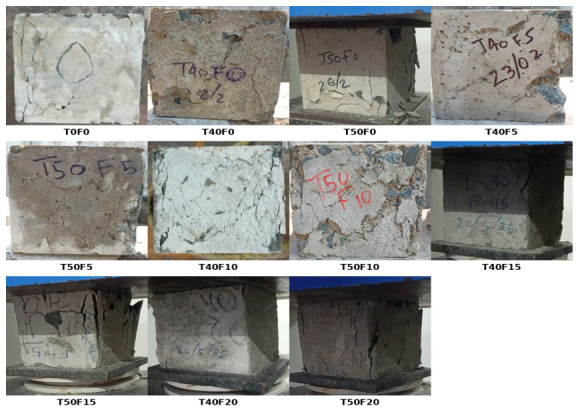
Failure surface characteristics of representative concrete mixtures after compressive strength testing.

SEM imaging of post-failure fracture surfaces was conducted to assess failure mode and paste–aggregate interfacial quality; this is distinct from the polished cross-section SEM–EDX microstructural analysis of hydration products reported in Section 3.7. The fracture surface morphology of representative concrete cubes after compressive strength testing is shown in Figure 6. The OPC control mixture (T0F0) exhibited a smooth, planar fracture surface with limited aggregate interlock, indicative of brittle failure along the weaker paste–aggregate interface. In contrast, the optimized mixture T50F10 produced a rough, tortuous fracture surface characterized by pronounced aggregate bridging and crack deflection, consistent with a tougher, more cohesive paste–aggregate interface resulting from ITZ densification via secondary GGBFS hydration and controlled sulfate activation [5,20]. As FGD gypsum content increased beyond the optimum, mixtures T50F15 and T50F20 exhibited progressively smoother fracture surfaces with visible macro-voids and reduced aggregate interlocking, confirming the matrix discontinuity identified by SEM analysis and consistent with the mechanical strength reductions reported in Section 3.2 and Section 3.5.

### 3.4. Strength Efficiency Index

**Figure 7:**
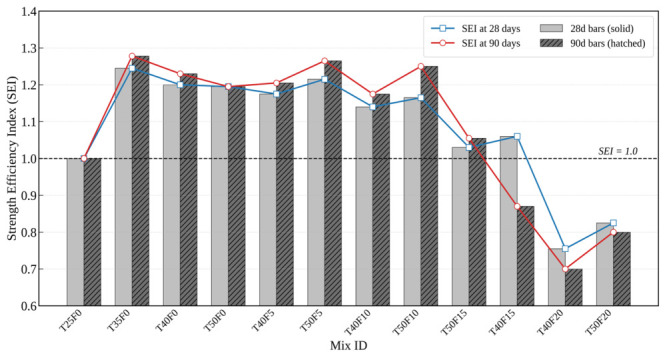
Strength efficiency index of all concrete mixtures at 28 and 90 days relative to the OPC control.

The SEI results at 28 and 90 days for all mixtures are presented in Figure 7. Within the binary GGBFS series, T25F0 recorded SEI ≈ 1.00 at both ages, indicating strength parity with the control, whilst T35F0 attained the highest binary SEI of 1.245 (28 d) and 1.278 (90 d). T40F0 and T50F0 returned 90-day SEI values of 1.233 and 1.200, respectively; the progressive increase from 28 to 90 days across all binary mixtures confirms continued slag hydration at later ages [5,21,37].

Among ternary blends, T50F5 recorded the highest 90-day SEI of 1.270, followed by T50F10 at 1.250; both exceeded all binary blends at 90 days except T35F0. T50F10 represents the optimal ternary composition when mechanical strength, durability and SEI are considered jointly, as discussed in Section 3.5, Section 3.6 and Section 3.7. At FGD, gypsum contents of 15–20%, SEI declined below or towards unity: T40F15 recorded 0.885 (28 d) and 0.870 (90 d), T50F15 remained marginally above unity at 1.030 (28 d) and 1.055 (90 d), and T40F20 and T50F20 returned 90-day values of 0.710 and 0.800, respectively. T40F20 is the only mixture in which SEI declined between 28 and 90 days (0.755 → 0.710), indicating that excess sulfate actively suppresses the long-term contribution of slag hydration rather than merely diluting clinker phases [15,19].

### 3.5. Tensile and Flexural Strength

**Figure 8:**
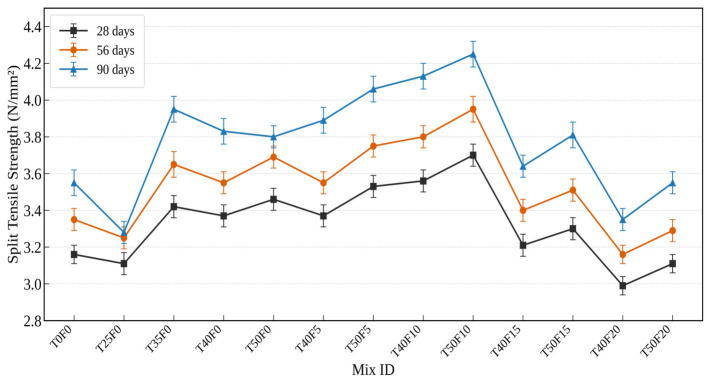
Split tensile strength development of OPC–GGBFS–FGD gypsum concrete mixtures at different curing ages.

**Figure 9:**
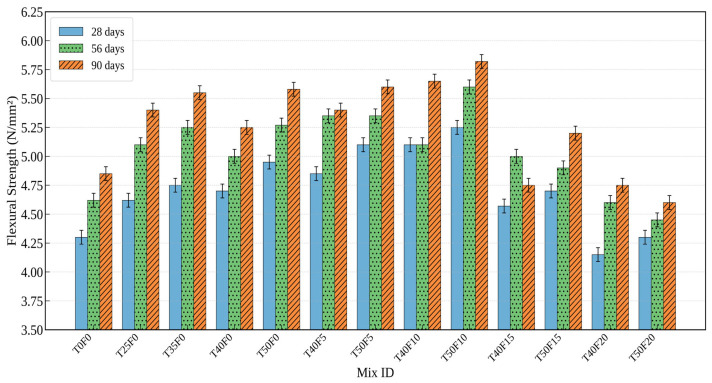
Flexural strength development of OPC–GGBFS–FGD gypsum concrete mixtures at different curing ages.

The split tensile strength and flexural strength results of all concrete mixtures at 28, 56 and 90 days are presented in Figure 8 and Figure 9, respectively. Both properties increased progressively with curing age, and the compositional trends were consistent across both test types, reflecting the common dependence of tensile and flexural failure modes on paste quality, pore refinement and aggregate–paste bond integrity.

Within the binary GGBFS series, T25F0 exhibited split tensile strengths slightly below the OPC control at all curing ages, whereas T35F0 recorded the most notable improvement, achieving split tensile strengths of 3.42, 3.65 and 3.95 N/mm^2^ at 28, 56 and 90 days, respectively. Flexural strength also increased progressively with increasing GGBFS content, with T50F0 recording the highest binary flexural strengths of 4.95, 5.27 and 5.58 N/mm^2^ at the corresponding curing ages. These improvements are primarily attributed to the gradual formation of secondary hydration products from GGBFS activation, which enhanced matrix compactness and aggregate–paste bond continuity [5,21].

The incorporation of moderate FGD gypsum contents (5–10%) enhanced both split tensile and flexural strengths relative to the binary blends and the OPC control. Among all ternary mixtures, T50F10 achieved the highest split tensile strengths of 3.69, 3.95 and 4.25 N/mm^2^ at 28, 56 and 90 days, corresponding to improvements of 16.8%, 17.9% and 19.4% over the control mixture. T50F10 likewise achieved the highest flexural strengths of 5.25, 5.60 and 5.82 N/mm^2^, representing a 20.0% improvement over the OPC control at 90 days. The enhanced performance of the ternary blends is attributed to sulfate-assisted slag activation and additional C–S–H formation, which refined the pore structure and strengthened the interfacial transition zone (ITZ) [5,20,34,36].

Beyond the optimum gypsum dosage of 10%, both split tensile and flexural strengths decreased progressively. T40F15 and T50F15 retained 90-day split tensile strengths of 3.64 and 3.81 N/mm^2^ and flexural strengths of 5.00 and 5.20 N/mm^2^, respectively, with both exceeding the OPC control at 90 days but declining substantially relative to T50F10. T40F20 and T50F20 recorded split tensile and flexural strengths at or below the OPC control at 28 and 56 days, with T40F20 recording the lowest tensile and flexural strengths of all tested mixtures. These reductions are consistent with increased matrix porosity, disrupted hydrate continuity, and micro-crack propagation arising from sulfate imbalance at excessive FGD gypsum dosages, as confirmed through SEM–EDX analysis discussed in Section 3.7 [15,19].

The tensile and flexural strength data collectively confirm that the optimum FGD gypsum content lies within the 5–10% range; beyond this threshold, the densification benefits of sulfate-activated GGBFS hydration are negated by progressive microstructural disruption from excess sulfate accumulation.

### 3.6. Durability Performance

#### 3.6.1. Rapid Chloride Penetration Test

**Figure 10:**
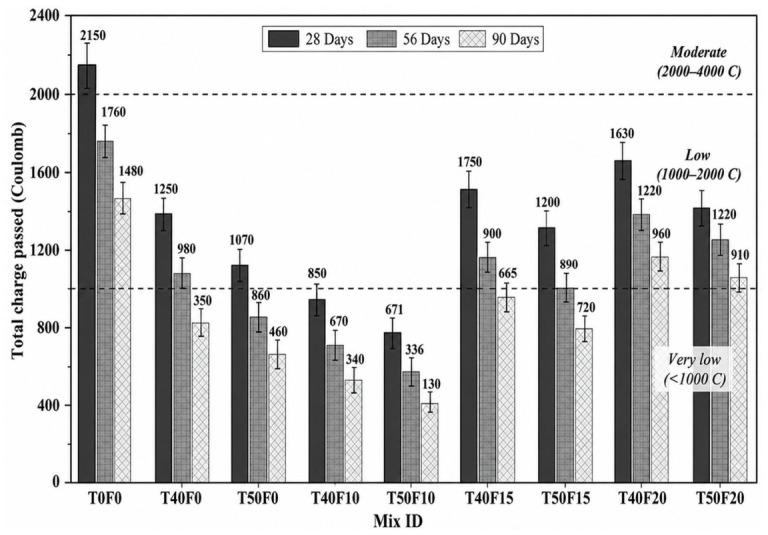
Rapid chloride permeability test (RCPT) results of OPC–GGBFS–FGD gypsum concrete mixtures at different curing ages.

The RCPT results at 90 days of curing for representative mixtures are presented in Figure 10 and summarized in Table 4. All mixtures exhibited a progressive reduction in total charge passed as curing age increased, confirming continued refinement of the pore structure from 28 to 56 to 90 days. The OPC control (T0F0) recorded approximately 1450 C at 90 days, classified as Low permeability per ASTM C1202 [32] (classification boundaries: Very Low < 1000 C; Low 1000–2000 C; Moderate 2000–4000 C). Binary GGBFS replacements progressively reduced charge passed; T40F0 and T50F0 recorded approximately 820 and 720 C, respectively, at 90 days, reflecting the well-established pore-refinement effect of slag secondary hydration [5,10,21]. T40F10 attained approximately 470 C, T50F10 attained a minimum of 410 C, a 71.7% reduction relative to the control, and T50F15 recorded 800 C despite its above-optimum gypsum content; all four mixtures fell within the Very Low permeability class (<1000 C). The reduction in total charge passed observed across the GGBFS- and FGD gypsum-containing mixtures reflects a combination of pore structure refinement from secondary hydration product formation and modifications to pore solution ionic composition arising from supplementary cementitious reactions [5,9,20,34]. These two contributions cannot be independently quantified from RCPT alone; the results are therefore interpreted as indicators of relative chloride penetrability rather than as direct measures of chloride diffusion coefficients.

Beyond the optimum range, RCPT values increased progressively with increasing FGD gypsum content. At 90 days, T40F15 (approximately 870 C) and T40F20 (approximately 1200 C) exhibited higher charge-passed values than T40F0 (approximately 820 C), while T50F20 (approximately 1060 C) exceeded T50F0 (approximately 720 C). These results indicate that excessive sulfate incorporation introduced matrix discontinuities that adversely affected chloride ion resistance. Similar trends have been reported in previous studies on gypsum-bearing blended concrete systems, in which elevated gypsum contents increased chloride penetrability by disrupting hydrate continuity [15,19].

#### 3.6.2. Water Absorption

**Figure 11:**
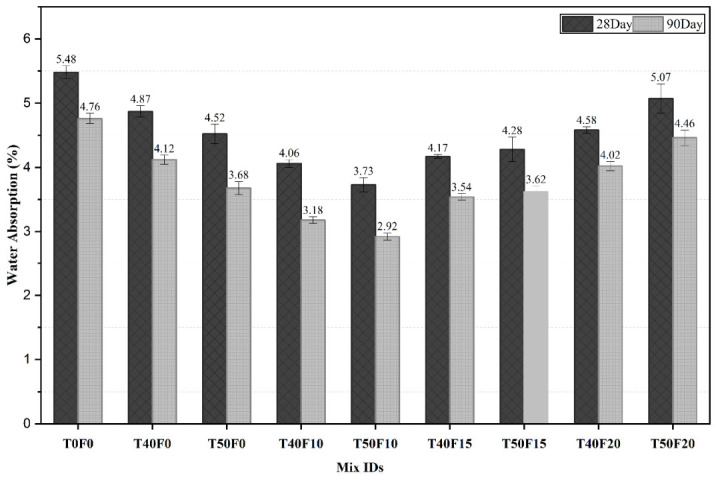
Water absorption values of OPC–GGBFS–FGD gypsum concrete mixtures at 28 and 90 days.

Water absorption at 90 days ranged from 2.92% (T50F10) to 4.76% (T0F0) among the representative mixtures (Figure 11). All mixtures exhibited a reduction in water absorption between 28 and 90 days, consistent with continued refinement of the pore structure through ongoing hydration reactions. Within the binary GGBFS series, T40F0 and T50F0 recorded 90-day water absorption values of 4.12% and 3.68%, respectively, representing reductions of 13.4% and 22.7% relative to the control (4.76%), confirming the pore-refinement effect of slag secondary hydration [5,21]. T50F10 exhibited the lowest water absorption at 90 days of 2.92%, a 38.7% reduction relative to the control, consistent with the dense, well-connected hydration product network observed in the SEM micrographs of this mixture. T40F10 recorded 3.18% at 90 days, confirming that the durability benefit of moderate FGD gypsum co-activation is not restricted to the 50% GGBFS level alone. Within the threshold zone, T40F15 and T50F15 recorded 90-day water absorption values of 3.54% and 3.62%, respectively, both below the control and indicating partial retention of the densification benefit despite an FGD gypsum content above optimum. The progressive increase in water absorption from the F10 to F20 series across both GGBFS replacement levels confirms the dilution and matrix discontinuity mechanisms identified in Section 3.2. T40F20 recorded 4.02% at 90 days, while T50F20 (4.46%) approached the control value (4.76%), and its higher within-group coefficient of variation relative to T50F10 reflects the microstructural heterogeneity characteristic of over-gypsified systems [15,19].

### 3.7. Microstructural Analysis

SEM–EDX analysis was performed on five representative mixtures at 90 days of curing to establish the microstructural basis for the mechanical and durability trends reported in Section 3.2, Section 3.3, Section 3.4, Section 3.5 and Section 3.6. Key quantitative results for these mixtures are summarized in Table 4. EDX provides semi-quantitative elemental composition at the point of measurement, reflecting bulk matrix chemistry rather than phase-pure compositions; apparent Ca/Si ratios are therefore reported as relative indicators of a hydration gel character rather than absolute hydrate descriptors. Given that these observations are based on single-specimen spot measurements across five representative mixtures, SEM–EDX findings are presented as corroborating evidence for the mechanical and durability trends reported in Section 3.2, Section 3.3, Section 3.4, Section 3.5 and Section 3.6, rather than as independent confirmation of hydration mechanism.

**Figure 12:**
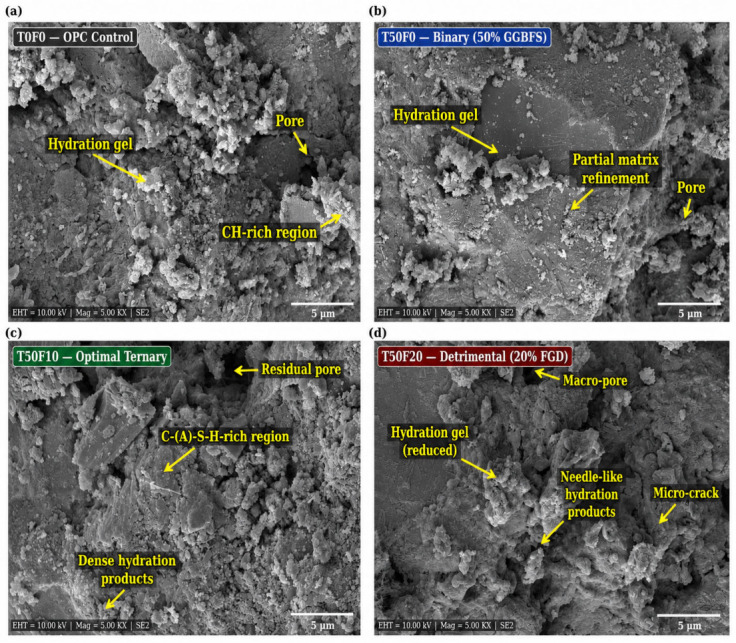
SEM micrographs of representative OPC–GGBFS–FGD gypsum concrete mixtures: (**a**) OPC control (T0F0), (**b**) binary GGBFS mixture (T50F0), (**c**) optimal ternary mixture (T50F10), and (**d**) detrimental mixture with excessive FGD gypsum content (T50F20).

**Figure 13:**
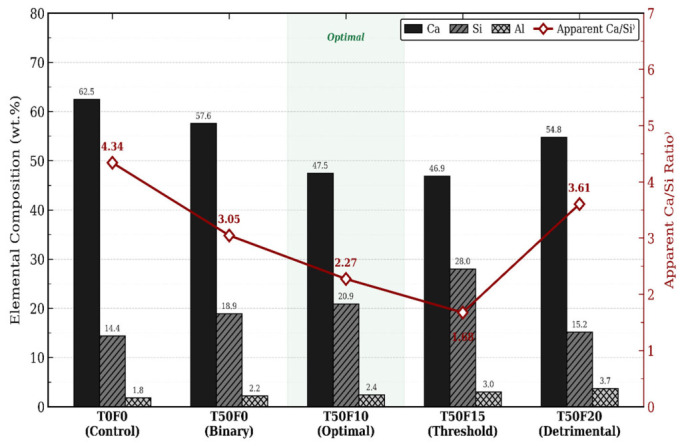
EDX elemental composition and apparent Ca/Si ratio of representative OPC–GGBFS–FGD gypsum concrete mixtures.

The SEM micrograph of T50F10 (Figure 12c) exhibited a dense, continuous binder matrix with well-distributed amorphous hydration products and minimal visible porosity. EDX analysis (Figure 13) yielded Ca = 47.5 wt.%, Si = 20.9 wt.% and Al = 2.4 wt.%, with an apparent Ca/Si ratio of 2.27. The elevated Si content and reduced Ca/Si ratio relative to the OPC control (Ca/Si = 4.34) and binary blend T50F0 (Ca/Si = 3.05) indicate a more polymerized C–(A)–S–H gel network arising from productive GGBFS dissolution under optimal sulfate conditions [20,34,38,39]. This microstructural observation is consistent with, and corroborates, the superior mechanical and durability performance of T50F10 reported across all tested parameters.

T0F0 (Figure 12a) displayed a portlandite-rich, coarse-pored matrix characteristic of OPC hydration without SCM contribution, yielding Ca/Si = 4.34 (Ca = 62.5 wt.%, Si = 14.4 wt.%). In T50F0 (Figure 12b), partial matrix refinement was evident, with the Ca/Si ratio reduced to 3.05 (Ca = 57.6 wt.%, Si = 18.9 wt.%), reflecting the onset of GGBFS-derived silicate gel formation in the absence of sulfate activation.

At the threshold FGD gypsum level, T50F15 yielded Ca = 46.9 wt.%, Si = 28.0 wt.% and Al = 3.0 wt.%, with an apparent Ca/Si ratio of 1.68. The Si content of 28.0 wt.%, the highest of all five analyzed mixtures, indicates extensive GGBFS dissolution; however, the degraded mechanical and durability performance reported in Section 3.2, Section 3.3, Section 3.4, Section 3.5 and Section 3.6 indicates that the silicate gel formed under excess sulfate conditions is of inferior structural quality, disrupting rather than reinforcing hydrate network continuity [15,19].

At 20% FGD gypsum content, SEM imaging of T50F20 (Figure 12d) revealed a discontinuous pore-rich matrix with macro-voids, micro-cracks and needle-like hydration products. EDX analysis yielded the highest Al content of all analyzed mixtures (3.7 wt.%), consistent with the availability of aluminate phases and the potential formation of sulfate-bearing aluminate hydrates, including ettringite-type phases, under conditions of excess sulfate supply [15,19,36]. The reversion of the apparent Ca/Si ratio to 3.61 (relative to 2.27 in T50F10) indicates a shift toward Ca-dominant, Si-depleted phase assemblages of limited mechanical contribution. Collectively, these observations are consistent with a transition from controlled sulfate-assisted densification to sulfate-induced matrix disruption at FGD gypsum contents exceeding 10% of total binder. The precise identification of the secondary phase assemblage responsible for the observed matrix disruption, including whether expansive ettringite formation contributes, would require complementary XRD and TGA analysis and is recommended as a focus of future investigation. Taken together, these microstructural observations, considered alongside the mechanical and durability trends reported in Section 3.2, Section 3.3, Section 3.4, Section 3.5 and Section 3.6, support the interpretation that the 10% FGD gypsum threshold reflects a genuine transition in hydration chemistry and matrix morphology rather than a statistical artifact of mechanical testing alone.

## 4. Conclusions

Based on the experimental investigation of thirteen OPC–GGBFS–FGD gypsum concrete mixtures at 28, 56 and 90 days of curing, the following conclusions are drawn:

- Untreated, as-received FGD gypsum from NTPC Singrauli is compatible with structural concrete production without requiring pre-treatment. All thirteen mixtures maintained slump values within 78–101 mm and fresh density within 2395–2420 kg/m^3^, confirming that workability and unit weight were not materially affected relative to the OPC control.
- Binary GGBFS replacement at 25–50% consistently improved long-term compressive strength beyond 14 days, driven by progressive C–S–H gel development from latent slag hydration. T35F0 attained the highest 90-day compressive strength of 55.6 N/mm^2^ (+33.7% relative to the OPC control), and all binary mixtures satisfied the M30 target mean strength requirement.
- The incorporation of 5–10% FGD gypsum by total binder mass further enhanced all mechanical and durability performance metrics relative to the binary blends and the OPC control. T50F10 achieved the highest ternary 90-day compressive strength (54.2 N/mm^2^), split tensile strength (4.25 N/mm^2^, +19.4%), and flexural strength (5.82 N/mm^2^, +20.0%), establishing the 5–10% FGD gypsum range as the optimum compositional zone across all test types and curing ages.
- FGD gypsum dosages exceeding 10% of total binder resulted in progressive deterioration across all six performance metrics at all curing ages. T40F20 and T50F20 recorded 90-day compressive strengths of 30.9 and 35.0 N/mm^2^, respectively, and failed to satisfy the M30 design requirement at 28 days, confirming a critical operational threshold beyond which FGD gypsum incorporation becomes detrimental to structural performance.
- The optimum composition T50F10 achieved a 90-day RCPT charge-passed value of 410 C (Very Low chloride ion penetrability per ASTM C1202 [32], −71.7% relative to the OPC control) and water absorption of 2.92% (−38.7%), representing the best performance among all evaluated mixtures on the durability indicators tested. The mechanical and durability optima coincided at T50F10 across all thirteen compositions, validating the 10% FGD gypsum threshold as a multi-parameter compositional boundary for both structural performance and transport-related durability criteria.
- SEI analysis confirmed superior clinker utilization efficiency in all GGBFS-containing mixtures within the optimum zone, with T35F0 (SEI = 1.278) and T50F10 (SEI = 1.250) recording the highest values at 90 days. T40F20 was the only mixture in which SEI declined between 28 and 90 days (0.755 → 0.710), indicating that excess sulfate supply suppresses long-term slag hydration rather than merely diluting the clinker fraction.
- SEM–EDX microstructural analysis at 90 days revealed a systematic reduction in apparent Ca/Si ratio from 4.34 (T0F0) to 2.27 (T50F10), consistent with increasing C–(A)–S–H gel polymerization under optimum sulfate activation. At T50F15, the highest Si content (28.0 wt.%) confirmed extensive GGBFS dissolution; however, the Ca/Si ratio of 1.68 and the concurrent reduction in mechanical and durability performance indicate that the silicate gel formed under excess sulfate conditions exhibited inferior structural quality. T50F20 exhibited a Ca/Si ratio of 3.61 with the highest Al content (3.7 wt.%) and needle-like hydration products, indicating a transition from sulfate-assisted densification to sulfate-induced hydrate disruption beyond the 10% threshold.
- It should be noted that this threshold was established for a single GGBFS source, a single untreated FGD gypsum source and a fixed water-to-binder ratio of 0.45; the transferability of the 10% threshold to other slag and FGD gypsum sources, mineralogies, impurity profiles, or water-to-binder ratios requires further validation.
- The durability assessment in this study was limited to chloride transport (RCPT) and water absorption; sulfate resistance, freeze–thaw resistance and long-term field performance were outside the present scope and are recommended for future investigation, particularly given the internal sulfate source introduced by FGD gypsum. Multi-objective optimization frameworks such as response surface methodology could also be applied in future work to simultaneously optimize mechanical performance, durability and CO_2_ reduction within the compositional window identified here.

Overall, the study demonstrates that controlled sulfate activation using untreated FGD gypsum can substantially enhance the performance and clinker efficiency of GGBFS-based structural concrete when maintained within a well-defined compositional window. The findings establish T50F10 as the optimum ternary blend and provide a practical framework for the sustainable utilization of industrial by-products in low-carbon structural concrete applications.

## Figures and Tables

**Table 1:** Physicochemical properties of constituent materials and aggregates: OPC, GGBFS and untreated FGD gypsum.

Property	OPC	GGBFS	Untreated FGD Gypsum
***Physical and particle-size properties***			
Specific gravity	3.09	2.70	2.04
D10 (µm)	2.81	7.5	2.6
D50 (µm)	~15 ^1^	40.0	11.7
D90 (µm)	~45 ^1^	150.0	51.0
***Chemical oxide composition***			
SiO_2_ (%)	21.30	34.85	3.12
CaO (%)	63.72	38.46	31.85
Al_2_O_3_ (%)	5.48	13.24	0.84
Fe_2_O_3_ (%)	3.92	0.76	0.41
MgO (%)	2.14	8.65	0.32
SO_3_ (%)	2.36	1.12	43.85
Loss on ignition (%)	1.96	1.42	12.74
Dominant phase	C_3_S/C_2_S	Amorphous glass	CaSO_4_·2H_2_O
***Aggregate properties***			
**Property**	**Fine Aggregate**	**Coarse Aggregate**	
Specific gravity	2.58	2.74	
Water absorption (%)	1.20	0.55	
Fineness modulus	2.75		
Nominal max. size (mm)	4.75	20	

^1^ OPC D50 and D90 adopted from IS 12269:2013 and published reference data. Chemical compositions obtained from supplier datasheets.

**Table 2:** Mix proportions of concrete mixtures (kg/m^3^).

Mix ID	OPC (kg/m^3^)	GGBFS (kg/m^3^)	FGD (kg/m^3^)	Water (kg/m^3^)	SP * (kg/m^3^)	Binder (kg/m^3^)	OPC/GGBFS/FGD (%)
**T0F0**	380	0	0	171	1.9	380	100/0/0
**T25F0**	285	95	0	171	1.9	380	75/25/0
**T35F0**	247	133	0	171	1.9	380	65/35/0
**T40F0**	228	152	0	171	1.9	380	60/40/0
**T50F0**	190	190	0	171	1.9	380	50/50/0
**T40F5**	209	152	19	171	1.9	380	55/40/5
**T50F5**	171	190	19	171	1.9	380	45/50/5
**T40F10**	190	152	38	171	1.9	380	50/40/10
**T50F10**	152	190	38	171	1.9	380	40/50/10
**T40F15**	171	152	57	171	1.9	380	45/40/15
**T50F15**	133	190	57	171	1.9	380	35/50/15
**T40F20**	152	152	76	171	1.9	380	40/40/20
**T50F20**	114	190	76	171	1.9	380	30/50/20

Fine aggregate = 710 kg/m^3^; Coarse aggregate = 1180 kg/m^3^; w/b = 0.45 for all mixtures. * Superplasticizer (PCE-based) at 0.5% of total binder mass (1.9 kg/m^3^). FGD and GGBFS percentages expressed as % of total binder mass (OPC + GGBFS + FGD = 380 kg/m^3^).

**Table 3:** Statistical treatment framework adopted for experimental data.

Parameter	Statistical Treatment
**Number of replicates**	3 specimens per mix per curing age for all mechanical tests (compressive, split tensile, flexural strength) and durability tests (RCPT, water absorption). SEM–EDX: single specimen per representative mixture; EDX spot analysis was not statistically replicated.
**Reported value**	Arithmetic mean ± 1 SD for all replicated tests. Error bars in all figures represent ±1 SD. SEM–EDX values represent single spot analysis measurements; no SD or error bars are reported for microstructural data.
**Variability indicator**	SD and CoV. CoV ranged from 1.33% (T50F10, 90 days) to 2.60% (T40F20, 14 days) across all 65 compressive strength test groups, with a mean CoV of 1.85%, confirming acceptable experimental reproducibility.
**Confidence level**	95%
**Outlier treatment**	Grubbs’ criterion (α = 0.05). No outliers were detected in any test group.

**Table 4:** Integrated durability and microstructural interpretation of representative concrete mixtures.

Mix ID	Zone	CS 90 d (N/mm^2^)	RCPT 90 d (C) [Class] ‡	WA 90 d (%)	Ca/Si †	Microstructural Interpretation
**T0F0**	Control	41.6	1485 (Low)	4.76	4.34	Portlandite-rich porous matrix; coarse pore network; high permeability relative to blended systems
**T50F0**	Binary	52.3	649 (Very Low)	3.68	3.05	Partial matrix refinement; moderate C–S–H gel development from slag hydration; reduced porosity
**T50F10**	Optimal	54.2	412 (Very Low)	2.92	2.27	Dense C–(A)–S–H-rich matrix with compact ITZ and refined pore structure
**T50F15**	Threshold	45.8	781 (Very Low)	3.62	1.68	Elevated Si content (28.0 wt.%); excess sulfate disrupts hydrate continuity despite continued GGBFS dissolution
**T50F20**	Detrimental	35.0	1064 (Low)	4.46	3.61	Discontinuous matrix; macro-voids; micro-cracks; needle-like sulfate-bearing products; Ca-dominant phase assemblage

† Apparent Ca/Si ratio is semi-quantitative; reflects bulk matrix EDX composition at 90 days, not absolute hydrate descriptors. ‡ RCPT permeability classifications per ASTM C1202: Very Low < 1000 C; Low = 1000–2000 C. CS = compressive strength; RCPT = rapid chloride penetration test charge passed; WA = water absorption; ITZ = interfacial transition zone; C–(A)–S–H = calcium (alumino-)silicate hydrate.

## Data Availability

The original contributions presented in the study are included in the article. Further inquiries can be directed to the corresponding author.

## Abbreviations

FGD	Flue gas desulfurization
GGBFS	Ground granulated blast-furnace slag
OPC	Ordinary Portland cement
SCM	Supplementary cementitious material
SEI	Strength efficiency index
RCPT	Rapid chloride penetration test
ITZ	Interfacial transition zone
CoV	Coefficient of variation
SD	Standard deviation
